# Highly reconfigurable oscillator-based Ising Machine through quasiperiodic modulation of coupling strength

**DOI:** 10.1038/s41598-023-31155-0

**Published:** 2023-03-10

**Authors:** Dagur I. Albertsson, Ana Rusu

**Affiliations:** Division of Electronics and Embedded Systems, KTH Royal Institute of Technology, Electrum 229, 164 40 Kista, Sweden

**Keywords:** Electrical and electronic engineering, Complex networks

## Abstract

Ising Machines (IMs) have the potential to outperform conventional Von-Neuman architectures in notoriously difficult optimization problems. Various IM implementations have been proposed based on quantum, optical, digital and analog CMOS, as well as emerging technologies. Networks of coupled electronic oscillators have recently been shown to exhibit characteristics required for implementing IMs. However, for this approach to successfully solve complex optimization problems, a highly reconfigurable implementation is needed. In this work, the possibility of implementing highly reconfigurable oscillator-based IMs is explored. An implementation based on quasiperiodically modulated coupling strength through a common medium is proposed and its potential is demonstrated through numerical simulations. Moreover, a proof-of-concept implementation based on CMOS coupled ring oscillators is proposed and its functionality is demonstrated. Simulation results show that our proposed architecture can consistently find the Max-Cut solution and demonstrate the potential to greatly simplify the physical implementation of highly reconfigurable oscillator-based IMs.

## Introduction

Unconventional computing paradigms based on natural processes have recently inspired the development of various hardware architectures, which can potentially outperform conventional Von-Neuman architectures for various applications, including machine learning^1,2^, chemistry^3,4^ and planning^5^. Ising Machines (IMs) belong to the class of architectures that employs the Ising Model for solving optimization problems. Optimization problems often rely on finding the global minimum in a multivariate energy landscape similar to the Ising Model. This analogy has been demonstrated by mapping various practically relevant optimization problems to the Ising Model^6^. Therefore, hardware architectures specifically designed to solve the Ising Model are considered as general purpose optimization solvers known as IMs.

The most widely known IMs are quantum annealers^7–9^, which are currently commercially available by D-Wave^10^. These systems are based on coupled Josephson junctions and have shown promising results as the first step to quantum computing^11^. For instance, the new D-Wave architecture includes more than 5000 qubits which in combination with their tools can solve optimization problems with up to a million variables^10^. However, quantum annealers operate at extremely low temperatures (sub Kelvin) requiring large cooling facilities and kilowatts of power, limiting the possibility of miniaturization.

Coherent Ising Machines (CIM)^12–14^ are based on degenerate optical parametric oscillators, which in combination with time-multiplexing allow for problems with thousands of variables to be solved^14^. However, CIM also have their specific challenges since they require long optical cables.

Digital CMOS IMs^15–22^, which are generally based on simulating systems that can solve the Ising Model, have been extensively studied in recent years. This approach comes with the advantages of using commercially available CMOS processes and consequently allow for rapid development and miniaturization.

Various analog based IMs^23–27^ have been also proposed. The proposal in^27^ utilizes coupled LC electronic oscillator networks for implementing an IM. This approach has recently been further investigated by the research community^28–34^ since it brings advantages including its potential for on chip implementation using CMOS technologies and low power consumption.

In recent years, emerging technologies, such as memristive^35,36^, p-bit^37,38^, spintronic oscillators^39–41^ and phase change oscillators^42,43^, have been also explored for implementing IMs.

A common design challenge affecting many of the previously discussed implementations is the number of coupling elements needed to implement IMs. Moreover, these couplings need to be highly reconfigurable to realize a general purpose IM. In this work, we investigate the possibility of implementing oscillator-based IMs with highly reconfigurable connectivity utilizing quasiperiodic modulation of the coupling strength. A similar approach has previously been explored for realizing oscillatory neuro computers^44–46^ and to achieve reconfigurability in quantum annealers based on Josephson parametric oscillators^47^. The proposed approach is analysed by using a network of Kuramoto oscillators. Further, a scaling scheme for large networks required for solving complex optimization problems is proposed. Finally, a proof-of-concept implementation based on CMOS RC ring oscillators is proposed and demonstrated.

**Figure 1:**
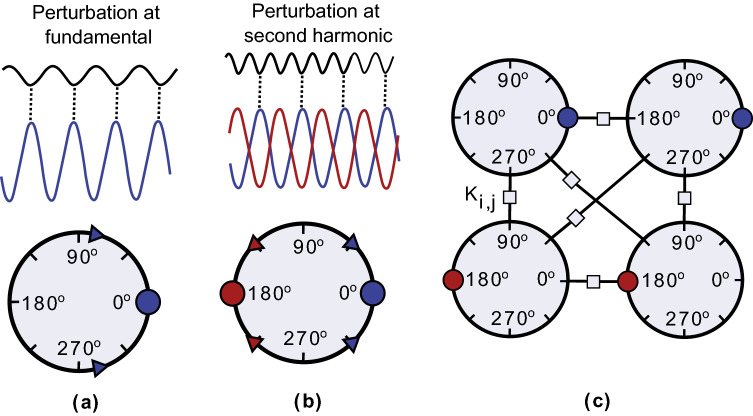
Graphical illustration of (**a**) fundamental injection locking, (**b**) SHIL and (**c**) a network of four coupled SHIL oscillators.

## Theory

**Figure 2:**
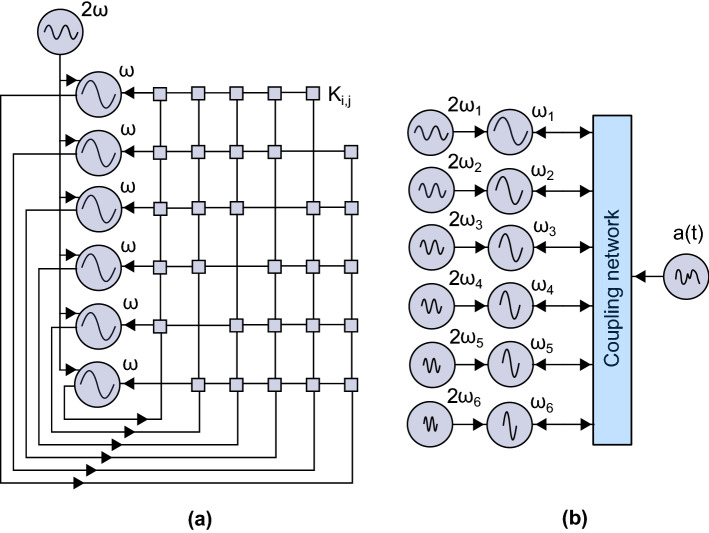
(**a**) A conventional oscillator-based IM requiring all-to-all coupling and (**b**) proposed implementation using a common medium.

The Ising Model describes a discrete magnetic system, where spins *s* settle to a binary state $\{+1,-1\}$. The Hamiltonian describing the energy of a spin configuration can be written as:

1 $$\begin{aligned} H(s) = -\frac{1}{2}\sum _{i,j = 1}^{N} J_{i,j}s_i s_j - \sum _{i=1}^{N}h_i s_i \end{aligned}$$

where N is the number of spins, $J_{i,j}$ is the coupling between spins *i*, *j* and $h_i$ is the interaction to an external field. However, for the following discussion, we consider $h_i = 0$. Oscillator-based IMs are based on the similarities between the Ising Model and the phase evolution of a synchronized oscillator network under second harmonic injection locking (SHIL), operating at the same frequency $\omega$. Considering a network of coupled Kuramoto oscillators^48^, the differential equations describing the phase evolution in a rotating frame $\theta _i(t) = \phi _i(t)-\omega t$ can be written as^27,49^:

2 $$\begin{aligned} \frac{d\theta _i}{dt} = - K_{s,i}sin(2\theta _i) -\sum _{j=1,j\ne i}^N K_{i,j} sin(\theta _i-\theta _j) \end{aligned}$$

where $\theta _i$ is the phase in the rotating frame, $K_{s}$ is the coupling strength to the SHIL, which is an externally applied signal at twice the fundamental frequency, $\omega _e=2\omega$, and $K_{i,j}$ is the coupling between oscillators *i*, *j*. The relation between (2) and the Ising Model in (1) can be understood through the graphical illustration presented in Fig. 1 (for simplicity, only four coupled SHIL oscillators are considered). When an oscillator is perturbed at a frequency equal to its operating frequency $\omega$, it approaches a phase locked state relative to the external signal, e.g. at $0^\circ$ as is highlighted by the arrows and dot in Fig. 1a. Similarly, if it is perturbed at twice the fundamental, two stable phase states appear at $0^\circ$ and $180^\circ$ (as in Fig. 1b), as a consequence of the term proportional to $sin(2\theta )$ in (2). In oscillator-based IMs, this bistability is used to represent the spin states in the Ising Hamiltonian (Eq. 1), where the oscillator settling to an odd/even multiple of $\pi$ represents a spin state of $+1/-1$. Finally, by coupling together a network of oscillators under SHIL, as presented in Fig. 1c, the phase dynamics become governed by (2) which has a global Lyapunov function equivalent to the Ising Hamiltonian^27^. Consequently, an oscillator-based IM can be realized with this relatively simple architecture. However, a major design challenge appears as the number of oscillators increases since N all-to-all connected oscillators require $O(N(N-1))$ coupling elements. It is worth mentioning that in Fig. 1c only $O(N(N-1)/2)$ bidirectional coupling elements are presented, but in circuit implementations $O(N(N-1))$ unidirectional coupling elements are generally needed. This is graphically illustrated in Fig. 2a where an architecture for 6 all-to-all connected oscillators is presented. Moreover, for a general purpose oscillator-based IM these coupling elements need to be highly reconfigurable allowing couplings to be turned-off, to have both positive and negative sign and even multilevel amplitude. Here, we will address the reconfigurability aspect by exploring one potential approach inspired from an oscillatory neurocomputer proposal^44^. Conventional oscillator-based IMs described by (2) are based on the assumption that all oscillators are synchronized and operate at the same frequency $\omega$. Consequently, in the rotating frame, the phase dynamics become governed by (2) which maps to (1) when the phases are binarized using SHIL. By substituting $\theta _i(t) = \phi _i(t) - \omega t$ into (2) and assuming a uniform coupling strength $K_{i,j} = K$ for all *i*, *j*, the phase dynamics in the stationary frame can be written as^50^:

3 $$\begin{aligned} \frac{d\phi _i}{dt} = \omega + K_{s}sin(2\omega t-2\phi _i)+K\sum _{j=1,j\ne i}^N sin(\phi _j-\phi _i) \end{aligned}$$

By extending this model to include oscillators operating at different frequencies ($\omega _i \ne \omega _j$), with the minimum difference between any *i* and *j* given by $\omega _{diff,min}$, and assuming that each oscillator *i* is perturbed by a separate second harmonic corresponding to twice its operating frequency $2\omega _i$, (3) can simply be written as:

4 $$\begin{aligned} \frac{d\phi _i}{dt} = \omega _i+ K_{s,i}sin(2\omega _i t-2\phi _i)+K\sum _{j=1,j\ne i}^N sin(\phi _j-\phi _i) \end{aligned}$$

By re-writing (4) in the rotating frame where $\phi _i(t) = \omega _i t +\theta _i(t)$ $(\phi _j(t) = \omega _j t + \theta _j(t))$ and $d\phi _i(t)/dt = \omega _i+d\theta _i/dt$, the phase dynamics become:

5 $$\begin{aligned} \frac{d\theta _i}{dt} = - K_{s,i}sin(2\theta _i)- K\sum _{j=1,j\ne i}^N sin((\omega _i-\omega _j)t+\theta _i-\theta _j) \end{aligned}$$

At this point, it is worth highlighting that each phase $\theta _i$ is in a different rotating frame since each oscillator operates at a different frequency $\omega _i$. Moreover, assuming that the coupling K in the network is weak ($K<<\omega _{diff,min}$) and constant, the phase dynamics are relatively unaffected by the second term in (5)^44^. Consequently, under these conditions, the oscillators can be considered to be uncoupled. However, by modulating the coupling strength *K* with a quasiperiodic function *a*(*t*), given by (6a), (5) becomes (6b):

6a $$\begin{aligned} a(t)= & {} \sum _{i=1}^N \sum _{j=1}^N c_{i,j}cos((\omega _j-\omega _i)t) \end{aligned}$$

6b $$\begin{aligned} \frac{d\theta _i}{dt}= & {} - K_{s,i}sin(2\theta _i)- Ka(t)\sum _{j=1,j\ne i}^N sin((\omega _i-\omega _j)t+\theta _i-\theta _j) \end{aligned}$$

When $(\omega _i-\omega _j) \ne (\omega _k - \omega _l)$ for all frequency differences in the system, the average phase dynamics of (6b) over a long time-span (proportional to 1/*K*) become:

7 $$\begin{aligned} \frac{d\theta _i}{dt} \approx -K_{s,i}sin(2\theta _i)-K \sum _{j=1}^N \frac{c_{i,j}+c_{j,i}}{2}sin(\theta _i-\theta _j) \end{aligned}$$

For full analysis of the averaging of (6b), the reader is referred to ’Supplementary material’. The average phase dynamics given by (7) closely resemble the original oscillator-based IM presented in (2). Consequently, a highly reconfigurable IM can be realized based on the modulation signal *a*(*t*), since the average coupling between oscillators in the network is purely determined by the coefficients $c_{i,j}$ in (6a). This proposal largely resembles the oscillatory neurocomputer explored in^44^, but a SHIL signal is additionally applied to all oscillators. In conventional oscillator-based IMs as it is presented in Fig. 2a, $O(N(N-1))$ coupling elements, connecting each oscillator to all others, are required. However, in the proposed implementation, oscillators are mutually connected to a single common coupling element that is modulated with $O(N(N-1))$ frequency components, as it is presented in Fig. 2b. This approach largely moves the complexity outside the oscillator network itself. Any arbitrary network can be realized by simply tuning the amplitudes $c_{i,j}$ of the modulation signals, which potentially allows for a scalable and flexible implementation. Nevertheless, the trade-off of this approach is that the phase dynamics evolve according to (7) on the slow time scale (proportional to 1/*K*), leading to slower convergence compared to conventional oscillator-based IM. However, the potential benefits of a greatly simplified implementation using this approach makes it worth exploring.

## Results

### Numerical simulations

**Figure 3:**
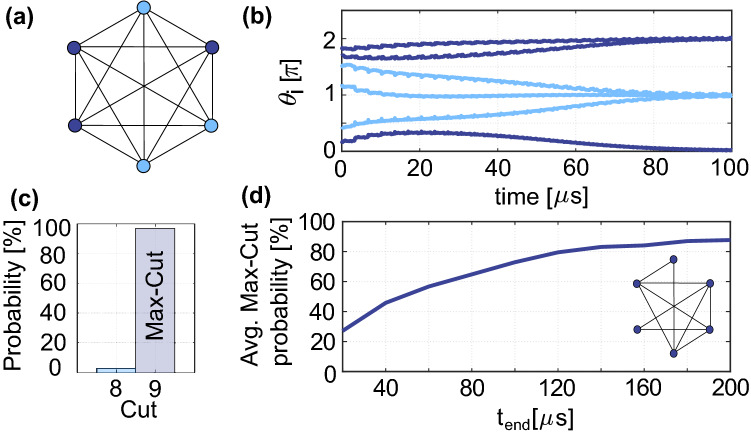
(**a**) An undirected unweighted graph of size N = 6 with all-to-all connections, (**b**) numerical simulations of the proposed oscillator-based IM, (**c**) solutions found for 100 independent simulations with random initial conditions, and (**d**) average max-cut probability for ten randomly generated graphs of size N = 6 (100 runs of each graph) for different simulation times $t_{end}$.

To proof that the system in Fig. 2b can be employed for developing an IM, we performed numerical simulations of (6) solving Max-Cut problems. The Max-Cut problem consists of partitioning the vertexes of a graph into two subsets $s_1$ and $s_2$, maximizing the number of edges crossing between the two sets. A simple graph example consisting of 6 all-to-all connected vertexes is presented in Fig. 3a. This simple Max-Cut problem is undirected ($c_{i,j} = c_{j,i}$) and unweighted (all edges have the same weight $c_{i,j}= 1$). The Max-Cut solution of this graph, consisting of three vertexes in $s_1$ while the other three in $s_2$, is 9. Any other solution than three in each set has a lower cut value (number of edges crossing between the two sets). To map this problem to an oscillator-based IM, vertexes represent oscillators and edges negative (antiferromagnetic) couplings. In our proposal, the antiferromagnetic (ferromagnetic) couplings are realized by setting $c_{i,j} = -1$ ($c_{i,j} = 1$), and $c_{i,j} = 0$ if two nodes do not share an edge. The frequency of the oscillators, $\omega _i$ (where $i = 1,2,\ldots ,6$), were chosen such that they form a Golomb ruler between $\omega _1 = 2\pi \cdot 5$ MHz and $\omega _6 = 2\pi \cdot 10$ MHz^45^:

8 $$\begin{aligned} \omega _i = \omega _{1}+\frac{\omega _{6}-\omega _{1}}{g_{6}}g_i \end{aligned}$$

where $\left[ g_1,g_2, g_3, g_4, g_5, g_6 \right] = [0,1,4,10,12,17]$ is the Golomb ruler. This approach maximizes the difference between any pairs of oscillators in the network, ($\omega _i-\omega _j$)^45^. Moreover, the coupling strength was chosen as $K \approx \omega _{diff,min}/20$, where $\omega _{diff,min} \approx 2\pi \cdot 0.3 MHz$, to achieve a weak coupling in the network, while the SHIL is ramped up as a function of time to binarize the phases in the system $K_s = (0.5 K \cdot t)/t_{end}$, where $t_{end}$ is the simulation time. Figure 3b shows the numerical simulation of the oscillator-based IM solving the graph in Fig. 3a, where the solution can be read by analyzing which oscillators settle to an odd/even multiple of $\pi$ at the end of the simulation. In this specific case, three oscillators settle to $\pi$ while three settle to $0/2\pi$ corresponding to the Max-Cut solution. Figure 3c presents the solutions found for 100 independent simulation runs with random initial conditions, showing a $96\%$ probability of finding the Max-Cut solution. To further verify that the couplings are determined by the modulation signal, we ran 100 simulations of 10 randomly generated graphs (total of 1000 runs) for various different simulation times. An example graph and the average probability of finding the Max-Cut solutions are presented in Fig. 3d. To map the random graphs to the oscillator-based IM, we simply turned off (by setting $c_{i,j} = 0$) the modulation signals corresponding to the absent edges in the graphs. The probability of finding the Max-Cut solution increases with the simulation time, which is generally also the case for conventional oscillator-based IMs. From the results presented in Fig. 3d, we can conclude that the approach presented Fig. 2b can be employed to realize a highly reconfigurable IM. Nevertheless, the need to distribute the operating frequencies according to a Golomb ruler for minimizing the unwanted couplings is a major disadvantage of this approach. As the number of oscillators increases, the operating frequency range $[\omega _{min},\omega _{max}]$ becomes impractical even for relatively small networks, limiting the experimental realization of oscillatory neurocomputers to small networks^45^. However, this approach is attractive for implementing IMs since IMs do not necessary require all-to-all connected networks as neurocomputers.

**Figure 4:**
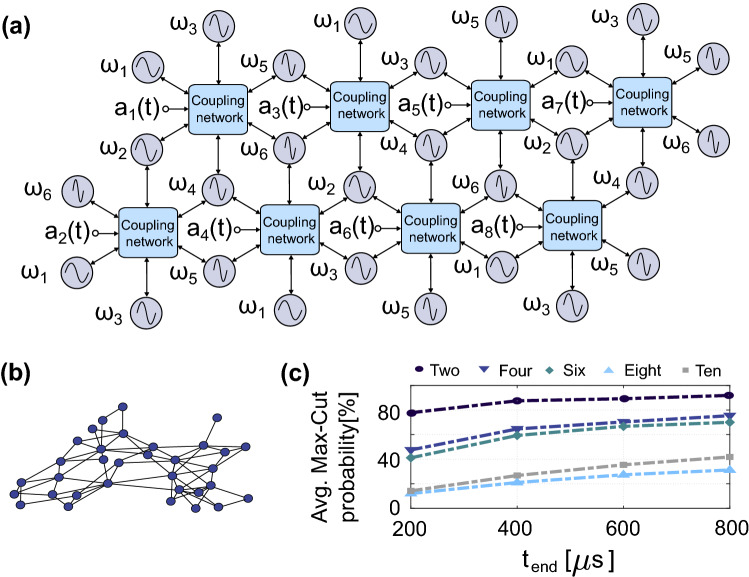
(**a**) Multiple hexagonal cells demonstrating a scalable implementation (note that the SHIL for each oscillator has been left out for clarity), (**b**) a randomly generated graph for ten cells and (**c**) average Max-Cut probability of randomly generated graphs for 2, 4, 6, 8 and 10 cells.

Our proposed architecture for implementing IM presented in Fig. 4a consists of hexagonal cells with six oscillators sharing a coupling network. This architecture allows for a scalable implementation, which brings the advantage of re-using operating frequencies of uncoupled oscillators. Moreover, the externally generated modulation signals can also be re-used between cells. However, the disadvantage of this approach is that each oscillator is now connected to twelve neighbouring oscillators (excluding boundary cases were it is less) instead of being all-to-all connected. This limits our implementation to graphs that can be mapped to the hexagonal grid. However, graph embedding techniques^51^ can potentially address this issue at the cost of additional pre-processing step and computational overhead^51,52^.

The functionality of the proposed architecture is demonstrated with numerical simulations. We generated ten random graphs for 2, 4, 6, 8 and 10 hexagonal cells and performed 100 simulation runs for each graph with random initial conditions. Figure 4b shows one of the ten graphs generated for ten hexagonal cells. Additionally, these simulations were performed for four different simulation times $t_{end}$ = [200  $\upmu$s, 400  $\upmu$s, 600  $\upmu$s, 800  $\upmu$s]. The Max-Cut for the ten random graphs (of each size) was found using LocalSolver, a commercially available optimization tool, and compared to the results of the numerical simulations for the proposed implementation. The resulting average Max-Cut probability for 100 simulation runs of each of the ten random graphs is shown in Fig. 4c. As expected, the probability of finding the optimal solutions decreases with shorter simulation time and for larger networks (higher number of cells). However, the proposed implementation is able to find the Max-Cut solutions consistently, especially for longer simulation times. Moreover, the amplitude of the SHIL signal is simply ramped in our simulations to phase binarize the system, but other schedules could greatly improve the probability of finding Max-Cut solutions^27^. However, the focus of this work is to demonstrate that the behaviour of the proposed system is dominated by (7) and that it is suitable to realize an oscillator-based IM. These simulation results further confirm that our proposal is a viable approach to realize highly reconfigurable oscillator-based IMs.

The implementation explored here is based on hexagonal cells consisting of six oscillators with operating frequencies distributed within $[\omega _{min},\omega _{max}] = 2\pi$ [5 MHz, 10 MHz]. In principle, the number of oscillators in a cell can be increased. However, the feasibility of doing this strongly depends on the practical implementation of the oscillator network. Specifically, the impact of increasing the number of oscillators has two implications for practical realization. First, for a certain frequency range or bandwidth, e.g. between 5 MHz and 10 MHz, the minimum frequency difference $\omega _{diff,min}$ between two oscillators in the network decreases, which makes the design of the oscillators more challenging. Secondly, the coupling K has to be smaller to satisfy $K<<\omega _{diff,min}$, which leads to longer convergence times.

### Proof-of-Concept Implementation using CMOS Ring Oscillators

**Figure 5:**
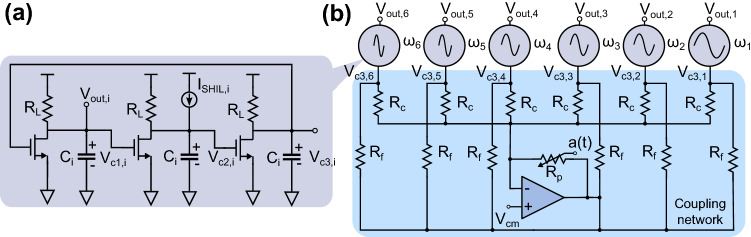
(**a**) A 3-stage SHIL RC ring oscillator and (**b**) a single hexagonal cell sharing a common coupling network consisting of six RC ring oscillators.

To demonstrate that the proposed approach can be implemented using relatively simple circuit elements, a proof-of-concept IM was developed in a 180 nm CMOS process using RC ring oscillators, as presented in Fig. 5. The implementation of a single cell (as it was presented in Fig. 2b) consists of six SHIL ring oscillators with load capacitances $C_i$ chosen to achieve operating frequencies $\omega _i$ ($i = 1,2,\ldots ,6$) distributed according to a Golomb ruler. The ring oscillators are coupled through a resistor $R_c$ to a summing amplifier and the tunable coupling element is implemented with a variable voltage controlled resistor $R_{p}$, as shown in Fig. 5b. The coefficients $c_{i,j}$ translate into the voltage amplitude of the modulation signal as $2c_{i,j}/(N(N-1))$ V. The resistance is modulated around zero and consequently requires both positive and negative values. In this proof-of-concept implementation, $R_p$ is modeled as an ideal voltage controlled resistor that can have both positive and negative values and is controlled by the modulation signal *a*(*t*). In a complete circuit implementation, this resistance can be implemented as in^45^ using a transistor in series with a negative resistance. The second harmonic injection locking in the ring oscillator shown in Fig. 5a is realized with a current source which is injecting a current at twice the fundamental:

9 $$\begin{aligned} I_{SHIL,i} = I_{inj}\frac{C_i}{C_6}\frac{t}{t_{end}}sin(2\omega _i t) \end{aligned}$$

**Figure 6:**
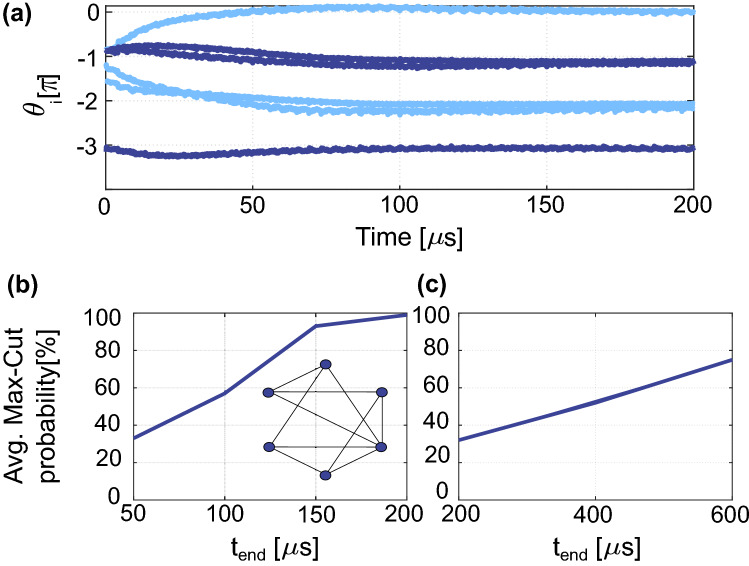
(**a**) Phase dynamics of a network of six all-to-all coupled ring oscillators solving the Max-Cut problem in Fig. 3a, (**b**) average Max-Cut probability for ten randomly generated graphs of size 6 (10 runs of each graph) for different simulation times $t_{end}$ and (**c**) average Max-Cut probability of randomly generated graphs mapped to 3 hexagonal cells (implemented with 13-stage RC ring oscillators).

The current is ramped up over the simulation time and normalized to the largest capacitance $C_6$, to achieve a uniform SHIL strength in all ring oscillators^53^. The advantage of the proposed implementation can be clearly observed since only a single tunable element is needed to set the coupling between any oscillators in the network. The differential equations describing the voltage on the capacitors can be written as:

10a $$\begin{aligned} C_i \frac{dV_{c1,i}(t)}{dt}= & {} \frac{V_{DD}-V_{c1,i}(t)}{R_L}-I_{D1,i} \end{aligned}$$

10b $$\begin{aligned} C_i \frac{dV_{c2,i}(t)}{dt}= & {} \frac{V_{DD}-V_{c2,i}(t)}{R_L}-I_{D2,i}-I_{SHIL,i} \end{aligned}$$

10c $$\begin{aligned} C_i \frac{dV_{c3,i}(t)}{dt}= & {} \frac{V_{DD}-V_{c3,i}(t)}{R_L}-I_{D3,i}-(V_{c3,i}-V_{cm})\Big [\frac{1}{R_c}+\frac{1}{R_f}\Big ]\nonumber \\&-\frac{R_p}{R_c R_f}\sum _{j=1}^N\frac{2a(t)}{N(N-1)}(V_{c3,j}-V_{cm}) \end{aligned}$$

where $I_{D1,i-D3,i}$ are the drain currents of the MOS transistors and $V_{cm}$ is the common-mode voltage of the summing amplifier. The coupling in the network is determined by the last term in (10c) where $K \propto R_p/(R_cR_f)$. For a full analysis of Eq. (10) and how it can be simplified to (7), the reader is referred to ’Supplementary material’. The phase of each oscillator relative to its SHIL signal was extracted by comparing the waveforms of the voltage $V_{c1,i}$ with a reference signal $V_{ref,i} = sin(2\omega _i t)$. To account for different couplings, as a consequence of the varying capacitance $C_i$, the $c_{i,j}$ were normalized to achieve a uniform coupling, similarly to what was done for the $I_{SHIL}$ in (9). Each SHIL RC ring oscillator was implemented with 13-stages (Fig. 5a shows only 3 stages for simplicity), $R_L = 5.75$ k$\Omega$ and a capacitance in the range from $C_1 = 482.5$ fF to $C_6 = 725$ fF resulting in $\omega _1 = 2\pi \cdot 10.75$ MHz and $\omega _6 = 2\pi \cdot 16.08$ MHz. The main motivation behind increasing the number of stages in the RC ring oscillators to 13 was to reduce the coupling in the network (which is proportional to the number of stages^53^) to satisfy $K<< \omega _{diff,min}$ and lower the operating frequency to tens of MHz. Moreover, the resistances $R_c = 25$ k$\Omega$ and $R_f = 4$ k$\Omega$ were also chosen to achieve a weak coupling in the network. Finally, the amplitude of the tunable resistance and the SHIL current are chosen as $R_p = 2 \mathrm{k} \Omega /\mathrm{V}$ and $I_{inj} = 10 \upmu$ A. To verify the functionality of the proposed implementation, transient simulations were performed and the phase difference was extracted. Figure 6a presents the phase difference between the oscillators and the reference signal $V_{ref,i}$ for an all-to-all connected graph of six oscillators settling to the Max-Cut solution. Figure 6b presents the average Max-Cut probability for 10 runs of ten randomly generated graphs for simulation times $t_{end}$ = [50  $\upmu$s, 100  $\upmu$s, 150  $\upmu$s, 200  $\upmu$s]. These simulation results confirm that the proposed implementation shown in Fig. 5 operates as an oscillator-based IM and consistently finds the Max-Cut solution.

To further verify that the proposed implementation can be scaled as shown in Fig. 4a, simulations of three coupled hexagonal cells were performed. It is important to note that the amplitude of oscillation needs to be kept relatively uniform throughout the network for a uniform coupling. Since oscillators on the boundary have different operating conditions (as a consequence of being only connected to one or two cells), these ring oscillators were appropriately designed to achieve a uniform oscillation amplitude. The simulation results, presented in Fig. 6c, demonstrate that the three hexagonal cells have also a typical oscillator-based IM behaviour.

## Discussion

A major challenge with realizing large scale IMs is the number of highly reconfigurable coupling elements that are needed. Consequently, the possibility of implementing a simple and tunable coupling on-chip could be one of the determining factors of what technology is best suited for realizing large scale IMs. On the algorithm site, various schemes to transform the Ising Hamiltonian from an all-to-all connected problem to a locally connected problem, such as minor embedding and LHZ, have been proposed^51,52^. However, these methods usually come with disadvantages such as computational overhead and performance degradation. On the hardware site, approaches to simplify the immensely complex hardware needed for IMs, such as time-division multiplexing^14^, computing in memory^18^ or using ferroelectric transistors to realize reconfigurable couplings^54^, have been explored. Oscillator-based IMs open the possibility to realize reconfigurability using different frequencies, similar to frequency division multiplexing in communication systems.

In this paper, we have proposed an oscillator-based IM, with high reconfigurability through quasiperiodic modulation of the coupling strength. Oscillators operating at distinct frequencies are mutually coupled through a common medium and connections are purely determined by the harmonic content of an externally applied modulation signal. Therefore, the proposed scheme could greatly simplify the implementation of highly reconfigurable oscillator-based IMs. The complexity is largely moved outside the oscillator network, specifically to the generation of the modulation signals. While a similar approach has previously been explored for Josephson parametric oscillator IMs^47^, this is the first work exploring oscillator-based IMs based on these principles, in CMOS technology. Moreover, although the implementation in^47^ shares many similarities with the approach proposed here, there is an important difference between the two approaches. Specifically, in^47^ the operating frequencies are distributed evenly with a unit difference. For this reason, mapping a problem to the architecture requires an additional computational step to correctly map a problem to the architecture (as is discussed in detail in^47^). In the approach proposed here, the need for this additional step is eliminated by a proper choice of the operating frequencies according to a Golomb ruler.

Additionally, to address the limitations of the proposed scheme, we investigated the possibility of using a hexagonal grid for a potentially scalable implementation. However, unless graph-embedding is combined with the hexagonal grid, the architecture is limited to relative sparse graphs. It is important to note that with currently available graph-embedding techniques, the computational overhead has the potential to diminish any advantages of hardware IMs^51,52^. Consequently, more research is required to evaluate the scalability of the proposed architecture for arbitrary dense graphs. Theoretically, the proposed approach can be combined with conventionally coupled oscillators operating at the same frequency. For example, long range interactions could be realized with conventionally coupled oscillators, while clusters of highly reconfigurable oscillators can be realized with the method proposed here. The oscillator-based IM has been also demonstrated with a proof-of-concept implementation based on CMOS RC ring oscillators. Nevertheless, the proposed approach comes at the cost of longer convergence times for finding the optimal solutions as a consequence of the weak coupling in the network. Quantifying this approach in relation to conventional oscillator-based IMs is challenging without exploring much larger benchmark graphs than presented here, which will be addressed in future work. It is important to mention that in^27^ a network of coupled LC oscillators has $K/\omega \approx 0.02$, while the proposed ring oscillator proof-of-concept has $K/\omega \approx 0.001$. Consequently, the phase dynamics in our proposed implementation takes place on a time scale $\approx 10x$ slower than the implementation in^27^.

Finally, the proposed approach is not limited to CMOS oscillators and can be applied theoretically to any oscillator having a sinusoidal coupling. Thus, it can potentially be explored for realizing novel oscillator-based IMs using emerging technologies, such as spintronic oscillator arrays^40,55^, where achieving highly reconfigurable coupling can be challenging. Moreover, an alternative approach, which it was not discussed in our manuscript, based on modulating the coupling signal of each oscillator separately^46,47^, could be also explored.

## Supplementary Information

Supplementary Information.

## Data Availability

The data used and/or analysed during the current study are available from the corresponding author on reasonable request.
