# Supplementary material for: Highly reconfigurable oscillator-based Ising Machine through quasiperiodic modulation of coupling strength
Source: Sci Rep. 2023 Mar 10;13:4005. doi: 10.1038/s41598-023-31155-0 (PMC10006240; doi:10.1038/s41598-023-31155-0)
Supplement: Supplementary file 1 — Supplementary Information. [file 41598_2023_31155_MOESM1_ESM.pdf]

Supplementary material: Highly Reconfigurable Oscillator-Based Ising Machine Through Quasiperiodic Modulation of Coupling Strength

Dagur I. Albertsson[1*] & Ana Rusu[1]

[1]*Division of Electronics and Embedded Systems, KTH Royal Institute of Technology, Electrum 229, 164 40 Kista, Sweden*

[*]*e-mail: dial@kth.se*

S1. Averaging the phase dynamics

Here we provide the full derivation of (7) from (6), similar to what was done in [1,2]. Starting from (6b):

$$\frac{d\theta_i}{dt} = -Ka(t)\sum_{j=1,j\neq i}^{N} sin((\omega_i-\omega_j)t+\theta_i-\theta_j) - K_{s,i}sin(2\theta_i) \tag{S1}$$

If K is small, we can write eq. (S1) as:

$$\frac{d\theta_i}{dt} = \sum_{j=1,j\neq i}^{N}\left(Kg_{i,j}(\theta_i,\theta_j)+\mathcal{O}(K^2)\right) - K_{s,i}sin(2\theta_i) \tag{S2}$$

where $g_{i,j}(\theta_i,\theta_j)$ is the average:

$$g_{i,j}(\theta_i,\theta_j) = \lim_{T\to\infty}\frac{1}{T}\int_0^T a(t)sin((\omega_j-\omega_i)t+\theta_j-\theta_i)dt \tag{S3}$$

The coupling function, which is given by:

$$a(t) = \sum_{k=1}^{N}\sum_{l=1}^{N} c_{k,l}cos((\omega_k-\omega_l)t) \tag{S4}$$

is written in terms of k,l instead of i,j for clarity, and it is inserted in (S3):

$$g_{i,j}(\theta_i,\theta_j) = \lim_{T\to\infty}\frac{1}{T}\int_0^T \sum_{k=1}^{N}\sum_{l=1}^{N} c_{k,l}cos((\omega_k-\omega_l)t)sin((\omega_j-\omega_i)t+\theta_j-\theta_i)dt \tag{S5}$$

which can be expanded as:

$$g_{i,j}(\theta_i,\theta_j) = \lim_{T\to\infty}\frac{1}{2T}\int_0^T \sum_{k=1}^{N}\sum_{l=1}^{N} c_{k,l}(sin((\omega_k-\omega_l)t+(\omega_j-\omega_i)t+\theta_j-\theta_i) - sin((\omega_k-\omega_l)t-(\omega_j-\omega_i)t-\theta_j+\theta_i))dt \tag{S6}$$

By choosing the frequencies ω_i such that all differences $(\omega_j - \omega_i)$ are distinct, the contribution from terms having a non-zero frequency component (i.e. when $k \neq j, i$ or $l \neq j, i$) averages out since the average of a sine over a period is zero. By writing out the sum in eq. (S6) and neglecting terms having a non-zero frequency component, (S6) simplifies to:

$$g_{i,j}(\theta_i,\theta_j) = \frac{c_{i,j}+c_{j,i}}{2}sin(\theta_j-\theta_i) \tag{S7}$$

which can be substituted back into eq. (S2) to arrive at eq. (7) in the main text (neglecting the term $\mathcal{O}(K^2)$):

$$\frac{d\theta_i}{dt} \approx -K\sum_{j=1}^{N}\frac{c_{i,j}+c_{j,i}}{2}sin(\theta_i-\theta_j) - K_{s,i}sin(2\theta_i) \tag{S8}$$

S2. Phase dynamics of an RC ring oscillator

Here we will show how the phase dynamics of a network of coupled RC ring oscillators can be reduced to a similar form of eq. (6) in the main text. Starting from eq. (9) in the main text and neglecting the SHIL current for simplicity:

$$C_i\frac{dV_{c1,i}(t)}{dt} = \frac{V_{DD}-V_{c1,i}(t)}{R_L} - I_{D1,i} \tag{S9a}$$

$$C_i \frac{dV_{c2,i}(t)}{dt} = \frac{V_{DD} - V_{c2,i}(t)}{R_L} - I_{D2,i} \tag{S9b}$$

$$C_i \frac{dV_{c3,i}(t)}{dt} = \frac{V_{DD} - V_{c3,i}(t)}{R_L} - I_{D3,i} - (V_{c3,i} - V_{cm})\Big[\frac{1}{R_c} + \frac{1}{R_f}\Big] - \frac{R_p}{R_c R_f} \sum_{j=1}^{N} \frac{2a(t)}{N(N-1)} (V_{c3,j} - V_{cm}) \tag{S9c}$$

Following a similar approach as in [3], we can re-write (S9) as:

$$C_i \frac{dV_{c1,i}(t)}{dt} = \frac{V_{DD} - V_{c1,i}(t)}{R_L} - I_{D1,i} - (V_{c1,i} - V_{cm})\Big[\frac{1}{MR_c} + \frac{1}{MR_f}\Big] - \frac{R_p}{MR_c R_f} \sum_{j=1}^{N} \frac{2a(t)}{N(N-1)} (V_{c1,j} - V_{cm}) \tag{S10a}$$

$$C_i \frac{dV_{c2,i}(t)}{dt} = \frac{V_{DD} - V_{c2,i}(t)}{R_L} - I_{D2,i} - (V_{c2,i} - V_{cm})\Big[\frac{1}{MR_c} + \frac{1}{MR_f}\Big] - \frac{R_p}{MR_c R_f} \sum_{j=1}^{N} \frac{2a(t)}{N(N-1)} (V_{c2,j} - V_{cm}) \tag{S10b}$$

$$C_i \frac{dV_{c3,i}(t)}{dt} = \frac{V_{DD} - V_{c3,i}(t)}{R_L} - I_{D3,i} - (V_{c3,i} - V_{cm})\Big[\frac{1}{MR_c} + \frac{1}{MR_f}\Big] - \frac{R_p}{MR_c R_f} \sum_{j=1}^{N} \frac{2a(t)}{N(N-1)} (V_{c3,j} - V_{cm}) \tag{S10c}$$

where M = 3 is the number of stages in the ring oscillator. This allows us to analyse only a single stage of the ring oscillator (we choose the 3rd stage here) and capture the overall behaviour. Since the coupling in the ring oscillator network is weak, we can analyse the system with a phase equation [2,4]:

$$\frac{d\phi_i}{dt} = \omega_i + Z_i(\phi_i) \frac{R_p}{MC_i R_c R_f} \sum_{j=1}^{N} \frac{2a(t)}{N(N-1)} (V_{c3,j}(\phi_j) - V_{cm}) \tag{S11}$$

where ω_i is the operating frequency and $Z_i(\phi_i) = \frac{\Delta\phi}{\Delta V}$ is the phase response function which describes the phase response of the RC ring oscillator per unit perturbation of voltage. Making the substitution $\phi = w_i t + \theta_i$ allows us to extract the phase dynamics as:

$$\frac{d\theta_i}{dt} = Z_i(\omega_i t + \theta_i)\frac{R_p}{MC_iR_cR_f}\sum_{j=1}^{N}\frac{2a(t)}{N(N-1)}(V_{c3,j}(\omega_i t + \theta_i) - V_{cm}) \tag{S12}$$

$Z_i(\phi_i)$ is extracted from numerical simulations where a single RC oscillator is perturbated by a current pulse I_{ext} and analysing the phase response [2]. The current pulse is kept very short, much shorter than the period of oscillations T, i.e. $\Delta t << T$ resulting in a voltage jump $\Delta V \approx \frac{I_{ext}}{C_i}\Delta t$. To calculate $\Delta\phi$, we simply analyse the period (between two zero crossing) where the pulse is applied T_e vs. an unperturbed period T_0 [2]:

$$\Delta\phi = 2\pi\left(1 - \frac{T_e}{T_0}\right) \tag{S13}$$

This is then repeated multiple times and the $\Delta\phi$ is analysed as a function of where in the period (0 to 2π) the pulse is applied. The phase response curve extracted by this method for $V_{th} = 414mV$, $C = 10pF$, $V_{DD} = 1.8V$, $R_L = 3k\Omega$, $1/2\mu_n C_{ox}\frac{W}{L} = 1mA/V^2$, $I_{ext} = 100\mu A$ and $\Delta t = 0.66ns$ is presented in Fig. S1. Here we can clearly see a phase shift of $\pi/2$ between the voltage amplitude and the phase response curve. Moreover, the RC oscillator output voltage and the phase response curve closely resemble a sine and cosine (as is highlighted with the fit) respectively. Consequently, we approximate $Z_i(\omega_i t+\theta_i) = Z_{p,i}cos(\omega_i t+\theta_i)$ and $V_{c3,j} = V_{cm}+V_{p,j}sin(\omega_j t+\theta_j)$ and eq. (S12) becomes:

$$\frac{d\theta_i}{dt} = Z_{p,i}cos(\omega_i t + \theta_i)\frac{R_p}{MC_iR_cR_f}\sum_{j=1}^{N}\frac{2a(t)}{N(N-1)}V_{p,j}sin(\omega_j t + \theta_j) \tag{S14}$$

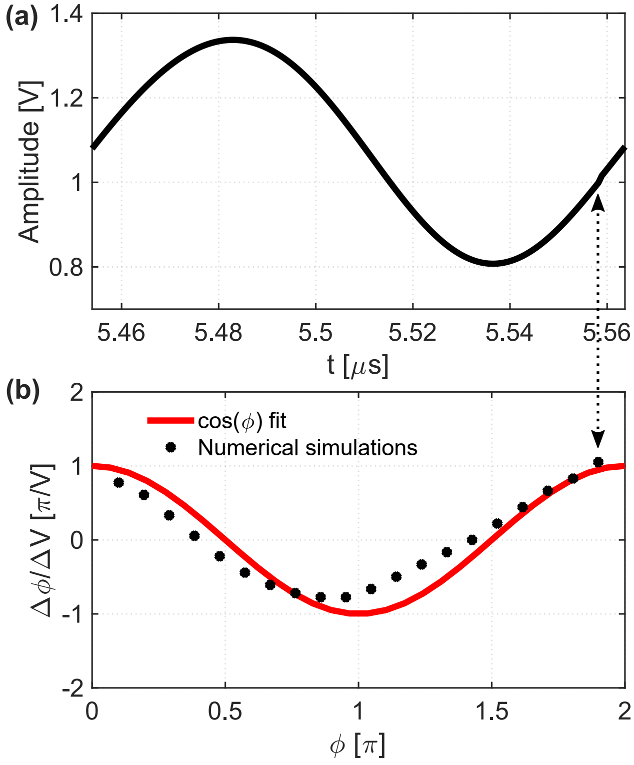

Figure S1: (a) Numerical simulation of a perturbated RC ring oscillator (only one period is presented) and (b) the phase response curve and a fit for $cos(\phi)$. The arrow highlights an example perturbation.

Assuming $Z_{p,i} = Z_p$ and $V_{p,j} = V_p$ (i.e. it is the same for all oscillators) and making the substitution

$$K_i = Z_p V_p \frac{2R_p}{MC_i R_c R_f N(N-1)}$$

eq. (S14) becomes:

$$\frac{d\theta_i}{dt} = -K_i a(t) \sum_{j=1}^{N} (sin((\omega_i - \omega_j)t + \theta_i - \theta_j) + sin(-(\omega_i + \omega_j)t - \theta_i - \theta_j)) \tag{S15}$$

which closely resembles the second term on the right of eq. (6b) in the main text, which is the starting point of the derivation in Section S1. The only difference is the second term on the right hand side of (S15). Performing a similar analysis as it was done in Section S1 shows that this terms

puts additional constraints on the choice of frequencies ω_i or that [2]

$$\omega_k - \omega_l \neq \omega_j + \omega_i$$

for all i, j, k and l. This analysis has previously been performed in [2] for a van der Pol oscillator. Lastly, a similar analysis can be performed for the second harmonic injection locking.

1. Hoppensteadt, F. C. & Izhikevich, E. M. Oscillatory neurocomputers with dynamic connectivity. *Phys. Rev. Lett.* **82**, 2983–2986 (1999). URL `https://link.aps.org/doi/10.1103/PhysRevLett.82.2983`.

2. Hölzel, R. W. & Krischer, K. Pattern recognition with simple oscillating circuits. *New Journal of Physics* **13**, 073031 (2011). URL `https://doi.org/10.1088/1367-2630/13/7/073031`.

3. Hazeri, A. R. & Miar-Naimi, H. Generalized analytical equations for injected ring oscillator with rc-load. *IEEE Transactions on Circuits and Systems I: Regular Papers* **65**, 223–234 (2017). URL `https://doi.org/10.1016/0304-4149(89)90040-9`.

4. Kuramoto, Y. *Chemical Oscillations, Waves, and Turbulence*, vol. 19 of *Springer Series in Synergetics* (Springer-Verlag, Berlin, Heidelberg, 1984). URL `https://doi.org/10.1007/978-3-642-69689-3`.
